# Assessing Functional Need of Minority Older Adults Across Two Long-Term Services and Supports Settings

**DOI:** 10.1093/geroni/igaa057.583

**Published:** 2020-12-16

**Authors:** Jasmine Travers, Andrew Cohen, Norma Coe, Mary Naylor

**Affiliations:** 1 Yale University, New York, New York, United States; 2 Yale University, New Haven, Connecticut, United States; 3 University of Pennsylvania, Philadelphia, Pennsylvania, United States; 4 NewCourtland Center for Transitions and Health, Philadelphia, Pennsylvania, United States

## Abstract

Research has suggested that growth in Black and Hispanic older adults’ nursing home (NH) use may be the result of disparities in options for long-term services and supports (LTSS). To investigate this issue, we aimed to determine whether there were no differences in the functional needs of racial and ethnic groups who received care in NHs versus the community. We identified respondents aged ≥65 years in the 2016 Health and Retirement Study who reported requiring caregiving help. We compared the site of care for Black and Hispanic older adults (minority group) to White older adults (comparison group). We performed unadjusted analyses to assess the association of functional need with community vs. NH care. Functional need was operationalized using a functional-limitations score and six individual activities of daily living (ADL). There were 186 minority older adults (community=78%, NH=22%) and 357 White older adults (community=50%, NH=50%). Across settings, minority older adults did not differ in age, marital status, and income, but a greater percentage of men were in NHs (48% versus 28%; p=0.01). The functional-limitations score was higher in NHs than in the community for both groups. Functional needs for the minority group were similar across the two settings in 2/6 ADLs (dressing p=0.11, toileting p=0.09), while White older adults in NHs were more impaired in all ADLs. Functional need for minority older adults primarily differed by setting while demographics did not. These are important factors to consider when implementing programs to keep older adults out of NHs and in the community.

